# Community-based interventions to enhance knowledge, protective attitudes and behaviors towards canine rabies: results from a health communication intervention study in Guangxi, China

**DOI:** 10.1186/s12879-016-2037-6

**Published:** 2016-11-24

**Authors:** Hairong Wu, Jiao Chen, Lianbin Zou, Liefeng Zheng, Weichao Zhang, Zhenmu Meng, Ricardo J. Soares Magalhaes, Youming Wang, Jingli Kang, Xiangdong Sun

**Affiliations:** 1 College of Journalism and Communication, Guangxi University, Nanning, Guangxi China; 2 Center for Animal Disease Prevention and Control of Guangxi, Nanning, Guangxi China; 3 Zhenmu Meng, Center for Animal Disease Prevention and Control of Baise, Baise, Guangxi China; 4 School of Veterinary Science, University of Queensland, Gatton, 4343 Australia; 5 Child Health Research Centre, University of Queensland, Herston, 4006 Australia; 6 China Animal Health and Epidemiology Centre, Ministry of Agriculture of the Peoples Republic of China, Qingdao, Shandong China

**Keywords:** Canine rabies, Health education, Intervention study, China

## Abstract

**Background:**

In China canine rabies poses a serious public health problem in that human mortality ranks the second highest globally. While rabies health education interventions are advocated by WHO to be critical components of modern rabies control and prevention programs, available studies have not adequately investigated the relative efficacy of their implementation in at-risk populations. This study aims to measure and compare the effect on knowledge and protective behavior towards rabies of health education interventions that include a novel Short Messaging Service via cell phone (SMS) and rabies health information sessions (IS).

**Methods:**

The study used a between-subject design involving repeated measures of rabies-related KAP (knowledge, attitude and practice). A total of 350 randomly selected villagers were randomly allocated into three intervention (SMS, IS and SMS + IS) and one control group. The content of SMS and IS covered topics about rabies prevention and route of transmission. The SMS intervention consisted of ten separate messages delivered three times two weeks after the pretest; the IS intervention was conducted once immediately after the pretest. A validated questionnaire was used to capture demographic information and KAP information. Ordinary Least Squares regression was used to contrast the effects of interventions.

**Results:**

Our results indicate that overall SMS outperforms IS at improving knowledge and protective behavior against rabies. Our results suggest that a combined intervention of SMS and IS can result in higher scores than any of the two in isolation. The impact of SMS, IS and SMS + IS is greatest on knowledge, followed by attitude and practice scores.

**Conclusion:**

This study demonstrated that health communication modes based on SMS, IS and a combination of the two are all effective to improve rabies-related KAP in the short term. These findings highlight the potential usefulness of SMS as an additional tool for public health communication and promotion; further studies are needed to investigate the long term benefits of these interventions on the reduction of dog bites and resulting human rabies incidence.

## Backgroud

Rabies remains the top neglect zoonotic disease worldwide; the global number of human rabies deaths in 2010 was estimated to be 61,000, corresponding to a total of 1.9 million disability-adjusted life-years (DALYs) lost at an estimated financial cost of US$ 6 billion [1].

In Mainland China canine rabies poses a serious public health problem in that human mortality of rabies is ranked the second highest in the world, just behind that in India. Currently in China, rabies ranks third in the list of 39 infectious diseases of mandatory notification in both 2012 and 2013 [2]. Recent studies have demonstrated that most rabies cases cluster in the provinces of the south of China [3] with reports indication a reemergence in Guangxi province [4].

Despite the recent efforts by the Chinese health authorities to improve the control and prevention of canine rabies, public health awareness on the disease is generally very low in areas most at-risk. For example, the results of a recent population-based survey in Hubei Province in China demonstrated, that only 31% of community inhabitants know that rabies virus may be carried by healthy dogs [4, 5]. The same survey has shown that after being bitten or scratched by animals, respondents did not consider the need to seek post exposure vaccination within 24 h after the event. These results highlight the need for population-based interventions aimed at increasing rabies-related knowledge towards improving attitudes and behaviors of at-risk populations, and the need to develop effective health education platforms to strengthen public awareness to reinforce rabies prevention.

The World Health Organization (WHO) has deemed health education and promotion as one of the three measures to prevent and control rabies, and maintained it the main strategy to alleviate the burden of disease worldwide in the future 10 years [6]. The first successful example of a national rabies health promotion campaign comes from India in 1985which gained broad support from the government, health care system and the community; ultimately, this lead to a sharp decline in rabies-associated mortality in the country [7]. More recently, studies from rabies endemic countries have highlighted the importance of appropriate rabies public awareness or health promotion campaigns to be part of routine rabies control plans [8–10].

Studies examining the effect of health education on rabies-related knowledge education or communication of the general populations have shown that health education interventions can improve respondents’ score of rabies-related knowledge, attitude and practice (KAP). While some studies focused on individuals with history of exposure to rabies at point of care [11–14], other studies targeted the general population with questions regarding preventive health behaviors [15–17]. However, the results of these studies need to be interpreted in light of critical methodological challenges. For example, confounding is an important determinant of the quality of the responses of participants with a previous history of canine rabies exposure. It has been reported that previous exposure can determine the level of response of individuals to a risk in that they tend to exacerbate risk and perceive a greater need for information [18, 19]. Consequently, those who have had exposure to rabies generally would have strong motivation for seeking health information compared to those not-exposed. Thus, in the case of studies focusing on individuals with previous history of rabies, the improvement of KAP scores after the education intervention may not entirely be attributed to the intervention. In addition, study design may also affect the observed results; to quantify the effect of health education interventions on rabies health prevention and knowledge five studies used a “before-after” design [12–15, 17] and two studies used an “after-only” design [11, 16]. The latter design can lead to effect sizes that could be confounded by the respondents’ prior knowledge or attitudes towards rabies prevention. In addition, while all seven studies used experimental designs, only two included a control group.

The communication methods for health education used in previous studies varied substantially and these included leaflets, billboards, lectures, outpatient counseling, sending out health education prescription supplemented by a video. Currently, SMS is one of most popular forms of communication in rural areas of China. A review of preventive health behaviors (e.g., smoking cessation) through the use of SMS suggests that SMS-delivered interventions can result in positive short-term behavioral outcomes [20]. Studies have demonstrated that SMS-based health promotion interventions may also be more cost-effective than interventions based on voice (e.g. telephone call) or print (e.g. leaflets/posters) [21, 22]. To date there are no studies on the effectiveness of SMS as a means to improve rabies health education, and there are no studies to contrast the effects between SMS and lecture-style information sessions.

This study aims to quantify the extent to which SMS and a rabies education information session (IS) can influence on KAP of at-risk populations, to determine the difference between SMS and IS on KAP of respondents and test whether a combined intervention of SMS and IS can achieve better KAP outcomes than any of two in isolation. The results are important to provide a basis for future health communication campaigns in China.

## Methods

### Ethics approval and consent to participate’ statement

Ethical approval for this study was provided by the Research Ethics Committee of Guangxi University. This analytical study utilized data of child participants and their parents/guardians provided informed consent on behalf of all child participants. All data used in the study were anonymised prior to analysis.

### Study design

The study was conducted in the county of Longlin, Guangxi Province of China from November 2011 to January 2012. Guangxi Province was selected because in the past five years it has consistently been the province with the highest incidence rate in China; in addition, recently there has been a reemergence of rabies cases in the province [4]. The Longlin County was selected because the incidence of human rabies is the second highest in Guangxi. In addition, the proportion of cell phone ownership in the Longlin County is approximately 90%.

**Fig. 1:**
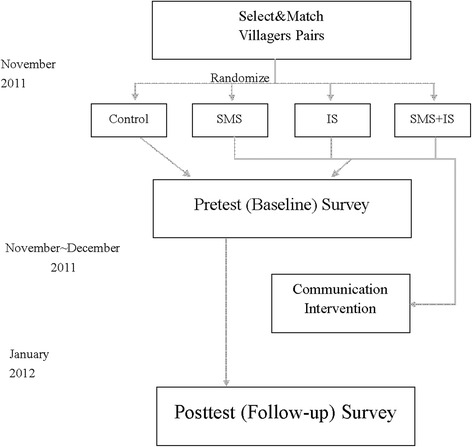
Trial Profile

The study used a between-subject design involving repeated measures of rabies-related KAP (Fig. 1). A total of 350 villagers from four villages of two townships subordinate to the county of Longlin were randomly allocated into three intervention groups (SMS, IS and SMS + IS) and one control group, using a multistage sampling procedure (Fig. 1). The inclusion criteria included participants above five years of age and owning a cell phone and the exclusion criteria included having a reported history of dog bites.

**Fig. 2:**
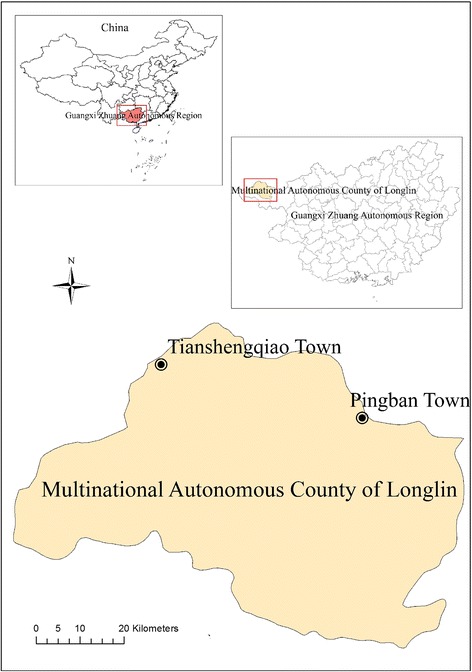
Map of the study area

The sample size and village selection was determined by a convenience sample that allowed striking a balance between the statistical power and study resources. We endeavored to control contamination between intervention arms by considering the selection of remote rural villages aided by the local knowledge of officials of Longlin animal husbandry and veterinary bureau. The two selected townships are approximately 500 km northeast of the capital city of Guangxi, located in Yungui highlands one of the poorest areas in Guangxi (Fig. 2). Village selection was also based on existing reports that these are important endemic foci for rabies in China [3] and on the suggestion from the officers of Longlin animal husbandry and veterinary bureau that these could represent typical rural communities.

The measurement tool used was a rabies knowledge, attitudes and practices (KAP) questionnaire; the questionnaire was delivered to all participants in each arm at baseline (before the intervention in the three intervention arms and at the beginning of the study in the control arm) and one month later. Questionnaires were filled out by participants and handed out to the research team upon completion.

### Interventions

Health education interventions tested in this study included: a) short message service via mobile phone (SMS), b) rabies education information session (IS), and c) a combination of the two (SMS + IS). SMSs were sent to participants of SMS and SMS + IS groups two weeks after the pretest. The SMS intervention included a total of ten different SMSs each with less than 60 Chinese characters; two different messages were sent every day and the ten SMSs were sent three times. The messages were based on the content included in the IS (Table 1). A 45-min IS was conducted immediately after the pretest covering topics on basic knowledge about rabies prevention, route of transmission, symptoms, present treatment and preventive measures as well as the rabies-relevant policy promulgated by the government.

**Table 1:** SMS messages sent as part of the intervention

Message One	Rabies is an infectious disease with about 100%mortality. That is to say, human rabies is incurable. Animals such as dogs, cats, bats and other wild animals can infect human beings with rabies.
Message Two	You can be infected with rabies if you are bitten or scratched by infected animals. Organ transplantation can also cause rabies if organ donor is infected.
Message Three	Once bitten by a rabid dog, a person will generally show symptoms after 20 to 90 days if they do not take vaccination. Bite site, depth and the size of wound can affect the length of the incubation period. The attack comes in a few days or after ten years.
Message Four	After being bitten by dogs, cats or other animals, you should immediately wash the wound thoroughly with soap or water for at least 15 min. After washing, clean the wound with iodine or alcohol. Do not bandage the wound. Rabies vaccine need to be taken within 24 h.
Message Five	There is a vaccine against rabies. The vaccine should be used five times. There is a fixed schedule. Once being bitten, you should take the rabies vaccine one dose at the time: on the day, and the third, seventh, fourteenth, twenty-eighth day after being bitten or scratched.
Message Six	Rabies treatment cost: The treatment includes injecting two kinds of medicine, the rabies vaccine and an immunoglobulin. It would cost approximately 1,750 Yuan for a 60 kg adult; price increases with weight.
Message Seven	Human health care centers/hospitals, veterinary clinics and centers for disease control can provide rabies vaccination to you. After completing the vaccination schedule, check again in a hospital to determine whether your body contains antibody for safety assurance.
Message Eight	In order to reduce rabies infection, dog owners must comply with the Domestic Dog Management Regulation”. Register your dog. Vaccinate your dog or cat once a year. Do not have close contact with dogs or cats^a^.
Message Nine	During the vaccination, do not take strenuous exercises, do not drink alcohol, tea or coffee; intense physical activity can increase secondary effects of rabies vaccination reactions
Message Ten	If you are bitten by a rabies vaccinated animal you also need to take the rabies vaccination as soon as possible because it is difficult to judge whether the animal is infected with rabies or not^b^.

^a^In rural area of China, the dog vaccination is far below 70% now. Given the situation, it is practical to discourage contacts with dog or cat too closely

^b^This message is based on the actual situation of dog vaccination and the report that normal dogs in Guangxi have a positivity rate of rabies virus of 3.26% (Re-emergence of Rabies in the Guangxi Province of Southern China. PLoSNegl Trop Dis 8(10):e3114.)

### Questionnaire on knowledge, attitudes and practices and scoring method

The KAP questionnaire was designed based on discussions with local experts on disease control and health communication and after consulting the peer-reviewed literature. The questionnaire included four sets of questions including basic demographic information, knowledge, attitudes and behaviors related to rabies.

A total of 16 questions on knowledge covered four topics including basic knowledge, rabies vaccination, wound treatments, disease prevention and treatment costs. Basic knowledge questions included questions such as “Is rabies an infectious disease?”. Questions about rabies vaccination were such as, “Is there a vaccine against rabies?”. Wound treatment included questions such as, “After being bitten by a dog, which of the following is the correct method of wound treatment?”. Questions about disease prevention and treatment cost were as follows: “Where will you seek treatment against rabies if you are bitten by an animal?” and “Do you know how much is the cost of the treatment required after being bitten by a dog (60 kg body weight for adults as an example)?”.

Eight questions on attitudes covered two topics including attitudes about prevention of rabies and dogs ownership. The first topic included questions such as “If you are bitten by an animal, would you get the injection of rabies vaccine within 24 h?”. The second topic covered questions such as, “Will you go to the management institution to register your dogs”.

Five questions on practices or behaviors included questions such as “Do you intend to keep cats, dogs or other animals continually?” and“Will you see a doctor if you are bitten by an animal?”

### Statistical analysis

A score was given to each part of the KAP questionnaire as follows: participants gained one point when they provided a correct answer to a question, and no points when the answer was wrong or the question was left unanswered. The total amount of points possible for the K part was 16, and the total possible score was 8 in the A part and 5 in the P part.

To contrast the effects of communication stimulus on participants’ rabies KAP, we used Ordinary Least Squares regression. This approach allows comparing the coefficients of the independent variables (i.e., exposure to the intervention) on the dependent variables (posttest KAP scores), while controlling for the effects of possible confounding variables. Regression analysis using the pretest score as a covariate can explicitly measure the impact of autocorrelation (i.e. the stability of KAP score), which is often the best explanation for the lack of stimulus effects. The general regression model for KAP score was expressed as:

$$ Pottest\; score=a+b(Exposure)+c\left( \Pr\;etes\; score\right)+d\left( Control \operatorname {var} iables\right) $$

Table 2 shows that, respondents’ sex, age, level of education and annual income are significantly different among the four groups. We could reasonably assume that these four variables may impact the posttest score. So the four variables were treated as covariates in our regression model. Thus, the following covariates were considered: age (with < 30 year of age group as the reference group); sex (with female as the reference group), education (with primary school and below as the reference group); annual income (with <1000Yuan as the reference group); pretest scores at baseline and intervention group (with control group as the reference group).

**Table 2:** Demographic characteristics of intervention and control groups

Variables	Control	SMS	IS	SMS + IS	*p-*value
Total number of participants	60	51	53	55	
Sex					
*Male*	47	21	37	29	<0.001
*Female*	13	30	16	26	
Age in Years					
*< 30*	15	9	5	15	0.038
*31–46*	27	16	30	23	
*> 46*	18	26	18	17	
Level of Education					
*Primary school and below*	49	11	34	8	<0.001
*Secondary school*	10	31	17	31	
*High school and above*	1	9	2	16	
Occupation					
*Planting*	35	33	25	32	0.216
*Livestock breeding*	4	6	10	4	
*Migrant workers*	6	8	6	8	
*Others*	15	4	12	11	
Annual Income in Yuan, RMB					
*< 1,000*	47	2	11	3	<0.001
*1,001–3,000*	11	22	33	13	
*3,001–6,000*	2	18	7	19	
*> 6,000*	0	9	2	20	
Having migrant workers in the family	35	27	32	41	0.121

To analyze the isolated marginal contribution of the sets of independent variable to the dependent variable, we calculated the partial coefficients of determination of each set of dummy variables according to the following formula.

$$ {R^2}_{y(klm)\cdot 1,2,\cdots, k-1}=\frac{RSS\left(1,2,\cdots, k,l,m\right)-RSS\left(1,2,\cdots, k-1\right)}{ESS\left(1,2,\cdots, k-1\right)}=\frac{{R^2}_{y\cdot 1,2,\cdots, k,l,m}-{R^2}_{y\cdot 1,2,\cdots, k-1}}{1-{R^2}_{y\cdot 1,2,\cdots, k-1}} $$

RSS: regression sum of squares

ESS: error sum of squares

In effect, four regression analyses and four calculations of partial coefficients were performed, each with one of the posttest scores, total KAP, knowledge scores, attitude scores and practice scores as the dependent variable. All analyses were conducted in SPSS 17.0.

## Results

### Data for analysis

At baseline, thirty-four respondents (10%; out of 350) left more than 15% of the questionnaire unanswered; therefore at baseline 316 questionnaires were useful for analysis. At follow-up, there were 272 questionnaires retrieved and 253 were deemed useful. As the sample was a panel one, after matching follow-up questionnaires to the baseline ones according to the demographic information including name, age, sex, level of education, the final useful sample size was 219, with 53 participants in the IS group, 51 participants in the SMS group, 55 participants in the SMS + IS group and 60 participants in the control group. Table 2 summarizes the demographic characteristics of individuals included in the analysis. The incomplete information results from 78 individuals absent at the follow-up survey and 53 individuals with incomplete questionnaires. We compared the demographic characteristics between individuals included in the analysis and individuals with missing/incomplete information and we did not find systematic differences. Similar losses for follow-up were identified for each of the intervention and control groups.

### Effect on general KAP scores

The effects of SMS + IS, SMS, IS, age, sex, level of education, annual income and baseline KAP scores on the follow-up KAP scores are shown in Table 3. Our results suggest that SMS + IS, SMS, IS, age (>46 years of age), sex (males), level of education, annual income (>6001Yuan) and baseline KAP scores are positively and significantly associated with follow-up KAP scores. As Table 4 shows, the partial coefficients of determination of communication stimulus ranks top among each set of independent variables.

**Table 3:** OLS coefficients about overall KAP, knowledge, attitude and practice scores

	KAP overall score		Knowledge score		Attitude score		Practice score	
Variables	Coefficient	*P-*value	Coefficient	*P-*value	Coefficient	*P-*value	Coefficient	*P-*value
Group								
*SMS + IS (vs Control)*	6.852	<0.001	3.366	<0.001	1.769	<0.001	0.336	0.038
*SMS(vs Control)*	4.819	<0.001	2.659	<0.001	1.023	<0.001	−0.020	0.896
*IS (vs Control)*	3.321	<0.001	1.797	<0.001	0.544	<0.001	0.217	0.121
Sex								
*Male (vs Female)*	1.856	<0.001	0.015	0.895	−0.040	0.534	0.106	0.579
Level of education								
*Secondary school* *(vs primary school or below)*	1.864	<0.001	1.127	<0.001	0.389	<0.001	0.731	<0.001
*High school and above* *(vs primary school or below)*	3.227	<0.001	2.507	<0.001	0.726	<0.001	0.916	<0.001
Age in years								
*31–45 (vs < 30)*	−0.195	0.437	0.108	0.464	0.117	0.162	0.117	0.345
*> 46 (vs < 30)*	−1.156	<0.001	−0.094	0.534	0.048	0.572	0.089	0.594
Annual income								
*1,001–3,000 (vs < 1,000)*	−0.353	0.282	−0.017	0.932	0.080	0.454	0.141	0.321
*3,001–6,000 (vs < 1,000)*	0.296	0.486	0.440	0.077	0.192	0.175	0.429	0.019
*> 6001 (vs < 1,000)*	1.215	0.023	0.961	0.003	0.500	0.004	0.807	<0.001
Pretest Scores.	1.031	<0.001	0.709	<0.001	0.716	<0.001	0.122	0.181
Intercept	−1.044	0.239	1.435	<0.001	1.223	<0.001	2.250	<0.001

**Table 4:** Partial coefficients of determination about overall KAP, knowledge, attitude and practice scores. The dependent variable is the post-test scores

Variables Set	KAP score	Knowledge score	Attitude score	Practice score
Communication Stimulus	0.574	0.513	0.516	0.051
Pretest Scores	0.496	0.353	0.462	--
Level of Education	0.225	0.319	0.108	0.176
Sex	0.216	--	--	--
Age	0.104	--	--	--
Annual Income	0.068	0.083	0.042	0.075

### Effect on individual knowledge, attitude and practice scores

Our results demonstrate that the effect of SMS + IS, SMS and IS on the posttest Knowledge and Attitude scores are statistically significant, after adjusting for level of education (i.e. Secondary school, High school and above), annual income (>6001) and pretest score (Table 3). Our results indicate that the effect of SMS + IS on the posttest Knowledge and Attitude scores is greater than SMS; our results also indicate that the effect of SMS on the posttest Knowledge and Attitude scores is greater than IS. Because the effect of sex, and age were not significant, the partial coefficients of determination of these two variables were not calculated.

Among all 12 independent variables, only 5 variables influence the posttest Practice scores significantly (Table 3). They are SMS + IS, Secondary school, High school and above, annual income (3,001-6,000) and annual income (>6,000).

The rank of partial coefficients of determination of each set of independent variables for each KAP component is shown in Table 4. In the Knowledge and Attitude components, communication stimulus ranks top two, followed by the pretest scores. Our results also suggest that in the Knowledge component, the effect of communication stimulus is 1.45 times higher than that of the pretest scores, while in the Attitude component, the effect of communication stimulus is 1.12 times higher than that of the pretest scores.

In the Practices component, the impact strength of all significant variables is weak. The top partial coefficient of determination is only 0.176, belonging to the variables related to the “Education level”. In the Practices component, the partial coefficient of determination of the communication stimulus ranks last.

## Discussion

This study demonstrates for the first time that a health education package that includes SMS is an effective platform to improve the knowledge, attitudes and practices towards canine rabies in a high-risk area for canine rabies in China. With this regard this study is the first of its kind to suggest that SMS can effectively deliver the necessary behavior change to reduce dog bites and associated exposure to rabies virus in the general population; this study is also the first to contrast the differential effectiveness of SMS and rabies education information sessions on knowledge, attitudes and practices towards canine rabies.

This study has been designed to address methodological challenges faced by previous studies and for that reason presents a methodological advancement in a number of ways. First, we considered that the dependent variables should be measured both before and after information stimulus and we have adopted a between-subjects design, contrasting respondents exposed and not exposed to the communication stimulus. Second, the respondents are drawn from the general public at risk of rabies, not those exposed to rabies.

Knowledge, attitude, belief and practice surveys are a conventional method to evaluate the knowledge, attitude and practice of the public about a certain health risk [23–25]. KAP theory indicates that health information is the premise to build positive attitudes and beliefs, and thereby enabled shape healthy behaviors though improved knowledge. In the context of rabies control,three KAP surveys on canine rabies have previously been conducted in Sri Lanka, Tanzania and Nigeria [26–28]. These studies suggested that knowledge gaps, cultural beliefs and behavior patterns may pose barriers to successful rabies control. In our study, we used KAP surveys as a tool to measure knowledge communication effects by contrasting the rabies-related KAP measurement before and after interventions between control and intervention groups.

Overall our results show that health communication interventions including SMS, IS and a combination of the two are all effective to the improvement of rabies-related KAP. Our results indicate that overall SMS is better than IS at improving knowledge and protective behavior against rabies. A possible explanation is that the short messages have been read repeatedly after they reached the audience. Importantly, our results indicate that a combined intervention of SMS and IS can achieve better effects than any of the two in isolation. The result is in accordance with a study about quitting smoking, which reported that education intervention combining mass communication with interpersonal communication is most desirable to lead to expecting outcomes about behavior and attitude change [29]. Our results also indicate that the statistical power of health communication ranks the top among all the influential factors.

Our results also demonstrated that the impact of each health communication intervention on each of the three parts of KAP is different, in that most benefits were observed on the knowledge component followed by the attitudes component. Knowledge is a necessary, but not a sufficient condition for behavior change; indeed, a major challenge that requires further investigation and attention is that the intervention did not bring about improvements in the practice component of KAP. Available evidence indicates that among knowledge, attitude and practice, there exists a cause-effect relationship, but it does not happen inevitably and other factors may play a role at influencing attitudes and practices. Some studies reported that the cost of dog vaccination and sterilization, the inaccessibility of facilities and the lack of services that would enable community participation in rabies control could be the reasons to weaken the impact of knowledge on practices [26, 27]. We also found that while sex and age of study participants play an important role on the overall KAP score, age and sex are not independently associated with the three parts of KAP separately. This finding suggests that, after accounting for income and level of education, the association between individual KAP scores and rabies interventions is not confounded by age and sex differences. This indicates that the interventions tested can impact equally on the knowledge and attitudes towards rabies in different age and sex strata of the population.

The results of this study should be interpreted in light of the study’s limitations. First, we were only able to obtain complete information for 219 individuals from the 350 initially screened at baseline. Local health workers ascribed their inability to further participate in the study due to the inconvenience of meeting with the study team as a result of the weak local public transport system. Furthermore, we did not find a systematic difference between individuals included in the analysis and those lost for follow-up. Second, we measured only short-term effects (i.e., right after exposure to IS) and further studies with extended follow-up are necessary to understand the long term influence of the interventions tested in this study.

## Conclusions

In conclusion, the communication interventions based on SMS and IS tested in this study have demonstrated to be effective means to increase community-level knowledge and attitudes towards rabies protection in a highly endemic setting. Among the three methods of educational intervention tested in our study, the combined use of SMS and IS is the most effective at increasing KAP scores, compared to an approach that implements them separately. The results provide a scientific basis for the potential importance and usefulness of SMS as an additional strategy of rabies health communication; SMS-based health promotion can potentially be included as a tool for public health actions for prevention and control of rabies and eventually other diseases. Long term effects and benefits of such an approach need to be further evaluated.

## Abbreviations

DALYs: Disability-adjusted life-years
ESS: Error sum of squares
IS: Information session
KAP: Knowledge, attitude and practice
RSS: Regression sum of squares
SMS: Short Messaging Service via cell phone
WHO: World Health Organization
